# Combining Phenotypes of Nucleotide Excision Repair Pathway to Predict the Risk of Head and Neck Squamous Cell Carcinomas in a Chinese Population

**DOI:** 10.1155/2022/4959737

**Published:** 2022-09-07

**Authors:** Yiqian Liang, Ling Zhang, Zichen Chen, Jiayu Chen, Sui Fang, Ting Zhang, Yun Zhu, Jie Liu, Baiya Li, Yuan Shao, Juanli Xing, Shaoqiang Zhang, Peng Han

**Affiliations:** ^1^Department of Otorhinolaryngology-Head and Neck Surgery, First Affiliated Hospital of Xi'an Jiaotong University, Xi'an, Shaanxi 710061, China; ^2^Department of Nuclear Medicine, First Affiliated Hospital of Xi'an Jiaotong University, Xi'an, Shaanxi 710061, China; ^3^Department of Otorhinolaryngology-Head and Neck Surgery, Second Affiliated Hospital of Xi'an Jiaotong University, Xi'an, Shaanxi 710004, China

## Abstract

**Background:**

Nucleotide excision repair (NER) is pivotal in the development of smoking-related malignancies. Nine core genes (*XPA*, *XPB*, *XPC*, *XPD*, *XPF*, *XPG*, *ERCC1*, *DDB1*, and *DDB2*) are highly involved in the NER process. We combined two phenotypes of NER pathway (NER protein and NER gene mRNA expression) and evaluated their associations with the risks of the head and neck squamous cell carcinomas (HNSCCs) in a Chinese population.

**Methods:**

We conducted a case-control study of 337 HNSCC patients and 285 cancer-free controls by measuring the expression levels of nine core NER proteins and NER gene mRNA in cultured peripheral lymphocytes.

**Results:**

Compared with the controls, cases had statistically significantly lower protein expression levels of XPA (*P* < 0.001) and lower mRNA expression levels of *XPA* and *XPB* (*P* = 0.005 and 0.001, respectively). After dividing the subjects by controls' medians of expression levels, we found an association between increased risks of HNSCCs and low XPA protein level (*P*_trend_ = 0.031), as well as low mRNA levels of *XPA* and *XPB* (*P*_trend_ = 0.024 and 0.001, respectively). Subsequently, we correlated the two phenotypes and found associations between the NER mRNA and protein levels. Finally, the sensitivity of the expanded model with protein and mRNA expression levels, in addition to demographic variables, on HNSCCs risk was significantly improved.

**Conclusions:**

Combining two phenotypes of NER pathway may be more effective than the model only including one single phenotype for the assessment of risks of HNSCCs.

## 1. Introduction

Head and neck squamous cell carcinomas (HNSCCs) are among the most common malignancies worldwide, which originate in the epithelial cells of the mucosal linings of the upper airway and food passages (the oral cavity, oropharynx, hypopharynx, and larynx) [1–4]. In the United States, the estimated number of new HNSCC cases has been increasing from 59,260 in 2010 to 65,410 in 2019, according to the American Cancer Society [5–7]. In China, there were 74,500 new HNSCC cases in 2015, according to the Chinese Cancer Society [8]. It is well established that tobacco smoking and excessive alcohol use are major risk factors for smoking-related HNSCCs and prior high-risk human papillomavirus (HPV) infection for HPV-positive HNSCCs, especially for the oropharyngeal cancer in the western world [9–11]. Although these risk factors may contribute to HNSCCs, only a small fraction of those who have the history of smoking, excessive alcohol use, or HPV infection develop one of these cancers in their lifetime, suggesting that there may be heterogeneity in HNSCCs susceptibility [12, 13].

Numerous carcinogenic chemicals in cigarette smoke can cause damages to cellular DNA [14]. For example, one of these chemicals is benzo (a) pyrene diol epoxide (BPDE) which is found in cigarette smoke as well as in the environment as a result of fuel combustion. The BPDE can induce irreversible damage to DNA by forming DNA adducts to block transcription of essential genes [15, 16]. Several DNA repair processes have evolved to repair these damages, among which nucleotide excision repair (NER) is a major and well-studied one [16–19]. NER essentially uses nine core proteins (XPA, XPB, XPC, XPD, XPF, XPG, ERCC1, DDB1, and DDB2) to restore the damaged DNA to normal one in living cells [20–22]. Functional mutations in any of these proteins may lead to abnormal NER and subsequently increase susceptibility to cancers including cancers of skin, lung, head, and neck [23–27].

So far, several studies have been reported concerning the association between the polymorphisms (genotypes) in the NER pathway genes and risks of different cancers [28, 29]. In a Chinese population study, the results indicated that *ERCC1* rs2298881 CA variant genotype was associated with an increased gastric cancer risk [30]. And another Chinese population study showed that five SNPs in NER genes are correlated with neuroblastoma susceptibility [31]. Furthermore, the association between the NER gene polymorphisms and risks of HNSCCs has been reported as well [22]. The *XPA* rs1800975 23A>G has been reported to be associated with HNSCCs, and *XPD* rs13181 also has been associated with HNSCCs [32]. In another large case-control study, it is reported that *XPC* Ala499Val SNP was associated with the risk of HNSCCs [33]. However, the associations of these biomarkers with cancer risks would not be fully elucidated if these analyses only contain genotype data. Thus, it is necessary to evaluate the associations between NER phenotypes (mRNA and protein levels) and risks of HNSCCs. In a non-Hispanic white population study, patients with reduced expression levels of NER proteins were reported to have an increased risk of having HNSCCs than those with higher protein levels [34]. Later, the same group validates these results in a study with a much larger sample size and more NER proteins [35]. Until now, there is no study exploring the above associations in the Chinese population, in which the composition of HNSCCs is quite different from that in non-Hispanic white population. Specifically, oropharyngeal cancer accounts for the most of HPV-positive HNSCCs in the United States, and the HPV-positive oropharyngeal cancer cases in previous non-Hispanic white study were about 91.4% of all the oropharyngeal cancer cases, while in the Chinese population, the most cases of oropharyngeal cancer are HPV-negative, meaning they are primarily caused by cigarette smoking [35–39]. In addition, the etiology of smoking-related HNSCCs is different from that of HPV-positive HNSCCs [40–42].

Although the NER translational and transcript levels have been studied separately for the risks of HNSCCs in a non-Hispanic white population, the effect of a single expression level of the NER pathway is quite limited for the prediction of HNSCC risk. Therefore, it is necessary to combine both NER translational and transcript levels to predict risks of HNSCCs. Subsequently, in the current study, we first validate the association of NER proteins and HNSCC risks in a Chinese population. Then, we test the effectiveness of combining two NER phenotypes in the HNSCC risk prediction model.

## 2. Materials and Methods

### 2.1. Study Subjects

We recruited 337 HNSCC patients and 285 cancer-free controls from the First Affiliated Hospital of Xi'an Jiaotong University during the period between 2013 and 2018. The cases were selected based on the following criteria: newly diagnosed, histologically confirmed HNSCCs but with no other cancers. The controls were recruited among visitors accompanying patients to the First Affiliated Hospital of Xi'an Jiaotong University. They were biologically unrelated to the cases, frequency matched with cases by age and sex, and have no history of prior malignancies. The subjects included in currently study were all Chinese Han. A written informed consent was obtained from cases and controls. Participants who smoked more than 100 cigarettes during their lifetime were defined as ever smokers, of which those who had quit smoking at least one year were defined as former smokers and remaining was considered current smokers; others were considered never smokers. Participants who drank alcoholic beverages at least weekly for one year were considered as ever drinkers, of which those who had quit drinking for more than one year were considered as former and the remaining was defined current drinkers; others were defined never drinkers. Each subject donated a 15 ml blood sample. The HPV status of all subjects was tested by RT-PCR assay. In the previous study, the expression levels of NER proteins were not correlated with the HPV status in the non-Hispanic white population [35]. Since the number of HPV-positive HNSCC cases was extremely limited with only five cases identified as HPV-positive, we could not infer whether NER protein levels were correlated with the HPV status. Thus, HPV-positive HNSCC subjects were excluded to avoid further heterogeneity in this study. The study protocol was approved by the First Affiliated Hospital of Xi'an Jiaotong University Institutional Review Board.

### 2.2. Reverse-Phase Protein Lysate Microarrays

Details regarding the current methods have been reported previously [35]. In detail, we isolated T-lymphocytes from whole peripheral blood by Ficoll gradient centrifugation. Cellular proteins were extracted from the cells and prepared for the RPPA analysis. Serial diluted lysates applied to nitrocellulose-coated slides (Schleicher & Schuell BioScience, Inc., USA) by Aushon Arrayer (Aushon BioSystems, USA). Each sample containing the antigens (the NER proteins) to be detected was spotted in duplicate with additional positive and negative controls prepared from mixed cell lysates or dilution buffer, respectively. Each slide was probed with a validated primary antibody plus a biotin-conjugated secondary antibody. Mouse anti-goat or anti-rabbit polyclonal or anti-human monoclonal antibodies were used against XPA, XPB, XPC, XPD, and ERCC1 (Santa Cruz, USA); XPF (Abcam, USA); XPG (Protein tech, USA); and DDB1 and DDB2 (Invitrogen, USA). The arrays were incubated with individual antibodies for 1 h at room temperature. The secondary antibodies were added to the slides and incubated at room temperature for 30 min.

Signals were amplified using a Dako system according to the protocol as previously described [35]. We then incubated the slides with a secondary conjugated streptavidin for 30 min and observed the signals by DAB colorimetric reaction. The signals on the microarrays were processed using the Array-Pro Analyzer software (Media Cybernetics, USA) to determine spot intensity, which were then analyzed by a logistic model by the R package. A fitted curve was plotted with the relative log2 concentration of each protein on the *X*-axis and the signal intensities on the *Y*-axis using the B-spline model as previously described [33]. Protein concentrations were determined from the fitted curve for each lysate by the curve fitting and normalized by the median value for protein loading as described [43, 44]. The RPPA_CF is the correction factor in RPPA. Samples were considered as an outlier, if the correction factor was below 0.25 or above 2.5.

### 2.3. Quantitative Real-Time PCR

Details of this test have been reported previously. Briefly, the mRNA expression levels were examined using the total RNA with the TRIzol reagent (Invitrogen, Carlsbad, CA) by the ABI 7900HT Sequence Detection System (Applied Biosystems, Foster City, CA). The process of thermal cycling conditions was done as follows: 95°C for 5 min, followed by denaturation at 95°C for 15 s for 40 cycles, and annealing/extension at 60°C for 1 min. Each sample was analyzed in triplicate. The Ct value or threshold cycle was the PCR cycle at which a significant increase in fluorescent signal was first detected. The 18S expression was used as an internal control. The expression levels of NER genes relative to that of 18S were calculated by delta Ct (ΔCt). The ΔCt value was the Ct value of the target gene subtracted its Ct value of 18S. Therefore, the higher the ΔCt values, the lower expression levels of the target mRNA.

### 2.4. Statistical Analysis

The distribution of demographic variables was evaluated between cases and controls by the chi-square test. The differences in the relative expression levels of NER proteins and mRNA levels were compared by Wilcoxon rank-sum test between cases and controls.

The medians of expression values were used in the controls as the cutoff values for calculating crude odds ratio (OR) and their 95% confidence intervals (CI). The associations between expression levels of protein and mRNA and HNSCC risk were estimated by computing ORs and CIs from multivariate logistic regression models. Further stratification analyses were used to evaluate effect modification of related expression levels of NER protein and demographic variables. A multiplicative interaction was defined as when OR_11_ > OR_01_ × OR_10_, in which OR_11_ was the OR when both factors were present, OR_10_ was the OR when only factor 1 was present, and OR_01_ was the OR when only factor 2 was present [45].

To assess effects of two NER phenotypes (protein and mRNA) on HNSCC risk prediction, four risk models were constructed to examine the area under the receiver operating characteristic (ROC) curve (AUC): the baseline model including only demographic variables, the protein model including expression levels of proteins in addition to these demographic variables, the gene model including mRNA expression levels of the genes in addition to these demographic variables, and the combining model including the expression levels of mRNA and protein in addition to these demographic variables. All tests were two-sided, and *P* < 0.05 was considered significant. All statistical analyses were performed using the SAS software (version 9.4; SAS Institute, Inc., Cary, NC).

## 3. Results

### 3.1. Characteristics of the Study Population

The summary of the distributions of selected characteristics of cases and controls is presented in Table 1. There were no significant differences in the distributions of age and sex between cases and controls. The average age was 58.8 years for the cases (median, 58; range, 40-91) and 58.7 years for the controls (median, 59; range, 40-90). Of all the subjects, 66.2% of cases and 64.2% of controls were male, 56.7% of cases and 50.1% of controls were ever smokers, and 67.3% of cases and 61.7% of controls were ever drinkers. There were more current smokers (32.1%) and current drinkers (33.8%) in cases than in controls (17.5% and 31.9%, respectively). The case/control ratios in all subgroups are near 1 : 1, except for current smokers which is 2.16 : 1. The primary HNSCCs of 337 patients included the oral cavity (67, 19.9%), oropharynx (150, 44.5%), and hypopharynx and larynx (120, 35.6%).

### 3.2. Differences in NER Protein or mRNA Expression Levels between Cases and Controls

**Figure 1:**
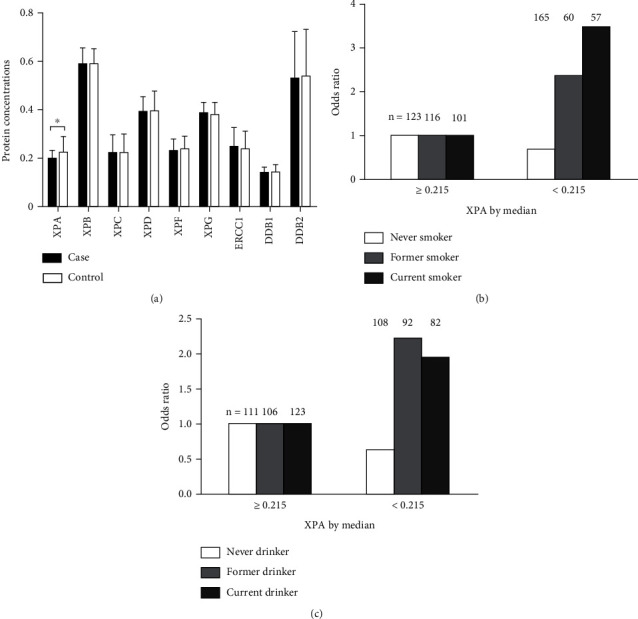
(a) Relative expression levels of nine NER proteins between HNSCC patients and healthy controls. Reverse-phase protein lysate microarrays were used to measure the relative expressions of nine NER proteins. (b) Modification effects of XPA by smoking status. (c) Modification effects of XPA by drinking status.

The cases showed lower relative mean expression levels in six of the nine core NER proteins analyzed than did controls, except for XPC, XPG, and ERCC1 (Table 2). In Wilcoxon rank-sum test for differences in NER protein expression levels between cases and controls, only XPA levels were statistically significantly lower in cases than in controls (*P* = 0.001; Figure 1(a)). Because the expression levels of the nine NER proteins were measured at the same time, they were likely to be correlated with each other. As shown in Supplementary Table 1, expression levels of XPA were statistically significantly correlated with XPB, XPC, XPD, and ERCC1 (*P* = 0.019, *P* = 0.050, *P* < 0.001, and *P* = 0.012, respectively). Moreover, mRNA expression levels of *XPA*, *XPB*, and *XPF* were statistically significantly lower in cases than in controls (*P* = 0.005, *P* = 0.001, and *P* = 0.035, respectively, Table 2).

### 3.3. Stratification Analyses of Expression Levels of XPA

Stratification analyses of XPA expression levels revealed that patients in subgroups of the age ≤ 59, age > 59, male, female, former and current smokers, and former and current drinkers exhibited significantly lower mean expression levels of XPA than did controls (all the *P* < 0.001, respectively, Table 3). In cases, women had lower expression levels of XPA than did men, but in controls, women had higher expression levels of XPA than did men, and the sex differences in the expression levels were insignificant in both case and control groups (*P* = 0.249 and *P* = 0.889, respectively, Table 3). Moreover, both ever smokers and drinkers had significant lower expression levels of XPA than did never smokers and drinkers, respectively (all the *P* < 0.001, respectively, Table 3). There were no significant differences in the expression levels of XPA by tumor sites, suggesting that expression levels of XPA may not be different among tumors of HNSCCs (Supplementary Table 2).

We further assessed possible interactions on a multiplicative scale between expression levels of XPA and selected variables listed in Table 1. The multiplicative interaction was tested when we included the interaction term (i.e., relative expression levels of XPA × each of the risk factors) in a multivariate regression model that also included the main effects of NER protein expression levels and other covariates. We found that smoking status as well as drinking status had significantly multiplicative interactions with relative expression levels of XPA (*P* = 0.005 and *P* = 0.044, respectively, Table 3), in association with HNSCC risk. To further unravel these multiplicative interactions, we stratified the adjusted ORs by smoking status and drinking status. It was apparent that ORs for the relative expression levels of XPA by median in groups of ever smokers were greater than those of never smokers (Figure 1(b)). And the ORs for the relative expression levels of XPA by medians in groups of ever drinkers were greater than those of never drinkers (Figure 1(c)).

### 3.4. Associations between Expression Levels of Protein and mRNA and Risks of HNSCCs

We first correlated expression levels of NER proteins with mRNA. As shown in Table 4, expression level of XPA was statistically significantly correlated with mRNA expression levels of *XPA* or *XPB* (*P* < 0.001 and <0.001, respectively). Then, to estimate HNSCC risks, the expression levels of proteins and mRNA were grouped into median values of the controls (Tables 5 and 6). The crude ORs for HNSCC risk associated with lower relative expression levels of XPA were 1.43 (95% CI, 1.04-1.97), compared with the high expression levels of XPA. After adjusting for age, sex, smoking status, and alcohol consumption in multivariate logistic regression analysis, the OR of XPA remained essentially unchanged. When continuous expression values were used in the logistic regression model with adjustment for all covariates, there was also a dose-response relationship between the reduced expression levels of XPA and the increased HNSCC risk (*P*_trend_ = 0.031). Furthermore, when continuous mRNA expression values were used in the logistic regression model with adjustment for all covariates, there were also dose-response relationships between the reduced mRNA expression levels and the increased HNSCC risks for *XPB* and *XPA* (*P*_trend_ < 0.001 and =0.024, respectively, Table 6).

### 3.5. Prediction of the Risk of HNSCCs by NER Protein and mRNA Expression

**Figure 2:**
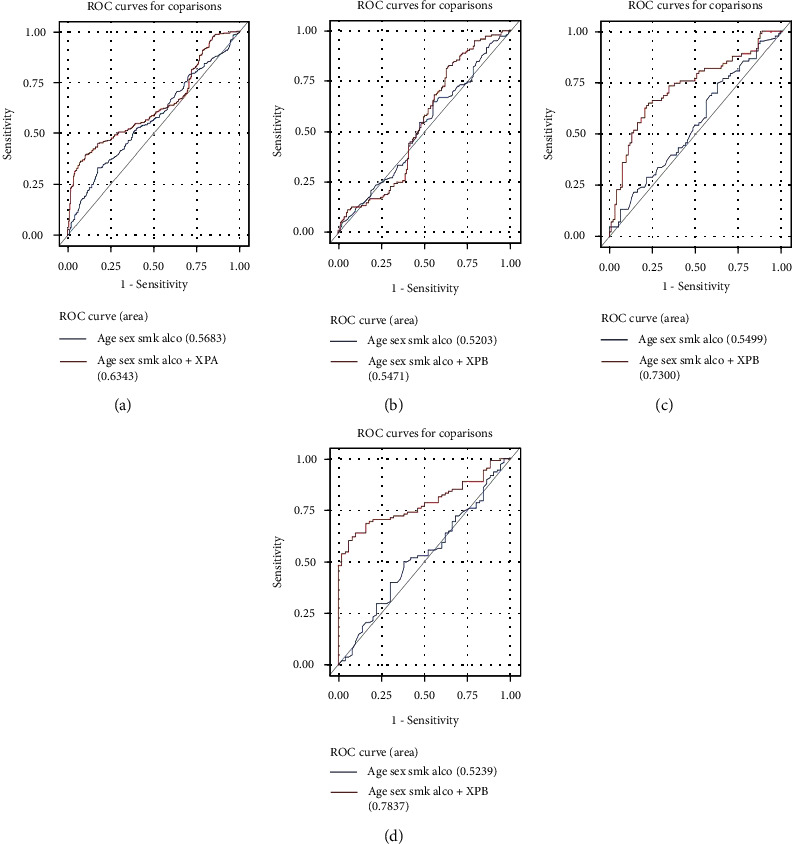
Overall and stratified ROC curves by smoking status calculated in multivariate logistic models. (a) The AUC was significantly improved in the model that included the effect of XPA expression levels, compared with the model that did not (*P* = 0.004). (b) The AUC was insignificantly improved in never smokers that included the effect of XPA expression levels (*P* = 0.462). (c) The AUC was significantly improved in former smokers (*P* < 0.001). (d) The AUC was significantly improved in current smokers (*P* < 0.001).

We assessed the performance of expression levels of NER protein on HNSCC risk prediction using the ROC curves. The AUC was significantly improved in the model that included the effect of XPA expression, compared with the model that did not (Figure 2(a), *P* = 0.004). Furthermore, the AUC was significantly improved in former and current smokers that included the effects of XPA expression, compared with the model that did not (Figures 2(c) and 2(d), *P* < 0.001 and *P* < 0.001, respectively), but insignificantly improved in never smokers (Figure 2(b), *P* = 0.462). The AUC was significantly improved in former and current drinkers that included the effects of XPA expression, compared with the model that did not (Supplementary Figure 1B and 1C, *P* = 0.001 and *P* = 0.001, respectively), but insignificantly improved in never drinkers (Supplementary Figure 1A, *P* = 0.404).

**Figure 3:**
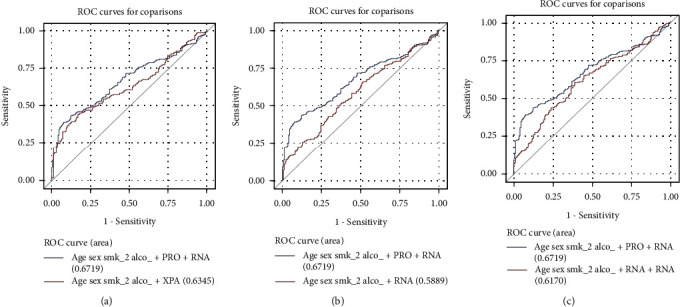
ROC curves in combined models. (a) The AUC in the ROC curves was significantly improved in the model including NER mRNA and protein (*XPA* and XPA, *P* = 0.010), compared to the model that included XPA alone. (b) The AUC was significantly improved in the model including NER mRNA and protein (*XPA* and XPA, *P* = 0.002), compared to the model that included *XPA* alone. (c) The AUC in the ROC curves was improved in the model including NER mRNA and protein (*XPA* and XPA, *P* = 0.056), compared to the model that included two mRNA levels (*XPA* and *XPB*).

We further assessed the prediction performance of models combining expression levels of NER mRNA and protein on HNSCC risks. Compared with the model that only included XPA, the AUC was significantly improved in the model including the effects of NER protein and mRNA (XPA and *XPA*; *P* = 0.010, Figure 3(a)). Besides, compared with the model that only included NER mRNA (*XPA*), the AUC was significantly improved including the effects of NER protein and mRNA (XPA and *XPA*; *P* = 0.002, Figure 3(b)). Compared with the model that included two mRNA levels (*XPA* and *XPB*), the AUC model was also improved including the effects of NER protein and mRNA (XPA and *XPA*; *P* = 0.056, Figure 3(c)).

## 4. Discussion

In this study, we firstly confirmed the results that reduced expression levels of NER protein were associated with an increased risk of HNSCCs in a Chinese population. Later, we assessed interactions between XPA expression levels and selected variables and found that smoking as well as drinking had significant multiplicative interactions with XPA expression on HNSCC risk. As the effect of a single phenotype in the NER pathway on the HNSCC risk prediction is quite limited, we combined expression levels of NER protein and mRNA in the ROC model. Our result showed that the model combining both NER protein and mRNA levels maybe more effective for the HNSCC risk prediction than the model that only included one phenotype.

In an early study, it was reported that there was an association between an increased risk of HNSCCs and reduced expression levels of XPD, XPF, XPA, and XPC in non-Hispanic white population, when appropriate antibodies for DDB1 and XPB were not available at that time [34]. Later, the same group validated the above results with more available antibodies for essential proteins and found the risks of HNSCCs associated with lower expression levels of XPA and XPB [35]. As the composition of HNSCCs in the Chinese population is quite different from that in non-Hispanic white population, we tested the associations between expression levels of nine core NER proteins and risk of HNSCCs in a Chinese population and found that the reduced expression levels of XPA was associated with HNSCC risk, but not for XPB. These results further support the notion that altered translational levels of NER pathway, which have a more direct effect on the NER capacity than that of transcript levels, may contribute to the risks of HNSCCs. Moreover, our previous work of transcript level suggested that mRNA expression levels of *XPA* and *XPB* were statistically significantly lower in cases than in controls, and the reduced mRNA expression levels of *XPB* were associated with an increased risk of HNSCCs in a Chinese population [46]. However, we did not find above association with *XPB* in translational level. One reason for this discrepancy is that the sample size of current study is still not large enough; future studies with more cases and controls are warranted to validate the current results. Another reason is that the transcript levels and translational levels of NER genes may not be directly correlated. Although the mRNA of NER gene is ultimately translated into a NER protein, the transcription and translation processes are far from a simple linear correlation [47]. The underling mechanisms are likely to be the cis-acting and trans-acting processes create a serial of systems that promote or inhibit the synthesis of proteins from a certain copy number of mRNA molecules, and translation levels are more directly involved in the NER repair process [48].

Previous study suggested a modification effect of smoking status on XPB, indicating that an association between the reduced expression levels of XPB and increased risk of HNSCCs may differ by smoking status [35]. In current study, we have observed smoking as well as drinking status had significant multiplicative interactions with XPA expression levels on HNSCC risk, other than XPB. Subsequently, we stratified the ORs of XPA by smoking and drinking status and found that the adjusted ORs for XPA in ever smokers or ever drinkers were greater than that in never smokers or never drinkers, indicating that ever smokers or ever drinkers might have a higher risk of developing HNSCCs with reduced XPA expression levels.

The XPA protein consists of several domains: the C-terminal domain is able to interact with the transcription factor IIH, the N-terminal domain with RPA34 and ERCC1 binding sites, and the central domain responsible for DNA binding [32]. Variation in XPA's functions may lead to an aberrant NER process and subsequently increase the susceptibility to cancer. Our data suggested an increased risk of HNSCCs associated with reduced expression levels of XPA in a Chinese population, and the current results were consistent with previously published non-Hispanic white studies on HNSCC risks, suggesting XPA may serve as a general biomarker for HNSCCs among two race groups.

Although the analysis of the NER mRNA expression is easy to perform, the results from the PCR assay may fluctuate in different cell stages. Thus, the data from transcript level maybe unsteady to predict HNSCC risks, and another assay is warranted to confirm the results from the PCR assay. The RPPA assay is a rapid, cost-effective, and most importantly an efficient method to measure the expression levels of NER proteins, and the current study is the first study to measure the associations between NER proteins and risks of HNSCCs in a Chinese population. Previously, we assessed the performance of NER proteins on HNSCC risk in the AUC model in a non-Hispanic white population and found that the AUC model was significantly improved by including the effects of XPB and XPA expression, compared with the model that did not [35]. In the current study, we found that the AUC model was significantly improved by XPA expression levels, suggesting that suboptimal XPA expression levels may play an important role in the risk of HNSCCs in two different races. Furthermore, we added NER mRNA expression in the protein-gene model and found the model that included NER protein and mRNA levels could significantly improve the AUC, compared with the model only included NER protein or mRNA alone. These results further implied that combining two phenotypes of NER pathway could significantly improve the effectiveness of the NER-HNSCC risk prediction.

Although this epidemiological study is the first large population study combining NER protein and mRNA levels to investigate risks of HNSCCs, there are still several limitations needed to be resolved. Like previous hospital-based studies, the control group may not be representative of the general population, and future studies may need a much larger sample size with more HPV-positive cases included and recruit the controls from the community-based population.

## 5. Conclusion

Reduced XPA expression levels were associated with an increased risk of HNSCCs in a Chinese population, and combining NER protein and mRNA may build a novel risk assessment model for HNSCC risk prediction.

## Figures and Tables

**Table 1:** Distributions of demographic variables and tumor characteristics between cases and controls.

Variable	Case (*n* = 337)	Control (*n* = 285)	Case/control ratio	*P* ^∗^
Sex				0.609
Male	223 (66.2)	183 (64.2)	1.22 : 1	
Female	114 (33.8)	102 (35.8)	1.12 : 1	
Age				0.484
Median (range)	58 (40-91)	59 (40-90)	0.98 : 1	
≤59	188 (55.8)	151 (53.0)	1.25 : 1	
>59	149 (44.2)	134 (47.0)	1.11 : 1	
Smoking				0.001
Never	146 (43.3)	142 (49.8)	1.03 : 1	
Former	83 (24.6)	93 (32.6)	0.86 : 1	
Current	108 (32.1)	50 (17.5)	2.16 : 1	
Drinking				0.331
Never	110 (32.6)	109 (38.3)	1.01 : 1	
Former	113 (33.5)	85 (29.8)	1.33 : 1	
Current	114 (33.8)	91 (31.9)	1.25 : 1	
Tumor site				
Oropharynx	150 (44.5)			
Larynx/hypopharynx	120 (35.6)			
Oral cavity	67 (19.9)			

HNSCCs = head and neck squamous cell carcinomas. ^∗^Chi-square tests for the distribution comparison of the demographic variables between cases and controls.

**Table 2:** Comparison of the expression levels of NER proteins and mRNA between cases and controls.

Genes	Protein (mean ± SD)		mRNA (mean ± SD)		*P*	*P* ^∗^
	Case (*n* = 337)	Control (*n* = 285)	Case (*n* = 221)	Control (*n* = 172)		
*XPA*	0.199 ± 0.033	0.225 ± 0.066	20.32 ± 2.29	19.76 ± 1.98	0.001	0.005
*XPB*	0.590 ± 0.066	0.591 ± 0.062	21.10 ± 2.08	19.34 ± 1.88	0.152	0.001
*XPC*	0.222 ± 0.075	0.222 ± 0.078	20.01 ± 1.98	20.90 ± 1.99	0.606	0.530
*XPD*	0.393 ± 0.061	0.395 ± 0.083	21.72 ± 2.14	21.33 ± 1.84	0.468	0.060
*XPF*	0.232 ± 0.047	0.239 ± 0.051	22.15 ± 2.02	21.70 ± 2.02	0.175	0.035
*XPG*	0.386 ± 0.045	0.381 ± 0.050	20.66 ± 2.00	20.53 ± 1.94	0.051	0.451
*ERCC1*	0.250 ± 0.077	0.239 ± 0.074	19.92 ± 2.20	19.92 ± 1.73	0.078	0.569
*DDB1*	0.142 ± 0.020	0.143 ± 0.028	18.49 ± 2.36	18.23 ± 2.05	0.630	0.785
*DDB2*	0.532 ± 0.192	0.540 ± 0.192	19.34 ± 2.06	19.29 ± 2.11	0.663	0.521

SD = standard deviation; IQR = interquartile range. *P* value in Wilcoxon rank-sum tests for proteins. The relative concentrations of each protein were normalized for protein loading and transformed to linear values. ^∗^*P* value in Wilcoxon rank-sum tests for mRNA. For the protein analysis, there are 337 cases and 285 controls. Among these subjects, 221 cases and 172 controls have NER mRNA data available.

**Table 3:** Stratification analyses of expression levels of XPA between cases and controls.

Variable	XPA (mean ± SD)		*P*∗	*P^*
	Case (*n* = 337)	Control (*n* = 285)		
Sex				0.951
Male	0.200 ± 0.032	0.223 ± 0.060	< 0.001	
Female	0.197 ± 0.035	0.227 ± 0.075	< 0.001	
*P*^∗^	0.249	0.889		
Age				0.196
≤59	0.200 ± 0.035	0.224 ± 0.065	< 0.001	
>59	0.197 ± 0.030	0.226 ± 0.067	< 0.001	
*P*^∗^	0.796	0.999		
Smoking				0.005
Never	0.211 ± 0.028	0.226 ± 0.065	0.683	
Former	0.192 ± 0.029	0.218 ± 0.046	< 0.001	
Current	0.187 ± 0.035	0.233 ± 0.093	< 0.001	
*P*^∗∗∗^	< 0.001	0.301		
Drinking				0.044
Never	0.206 ± 0.029	0.224 ± 0.074	0.490	
Former	0.198 ± 0.035	0.224 ± 0.040	< 0.001	
Current	0.193 ± 0.033	0.227 ± 0.074	< 0.001	
*P*^∗∗∗^	0.001	0.246		

*P* value in Wilcoxon rank-sum tests. *^^^P* value in multiplicative interaction analysis between selected variables and proteins in relation to HNSCC risk. The relative concentrations of each protein were normalized for protein loading and transformed to linear values.

**Table 4:** Correlation between NER proteins and mRNA expression levels.

Proteins/genes	Spearman correlation coefficients/*P* value								
	*XPA*	*XPB*	*XPC*	*XPD*	*XPF*	*XPG*	*ERCC1*	*DDB1*	*DDB2*
XPA	-0.255	-0.223	-0.072	-0.074	-0.052	-0.011	-0.068	-0.054	0.001
	< 0.001	< 0.001	0.157	0.144	0.301	0.821	0.176	0.282	0.993
XPB	-0.001	-0.013	0.174	0.060	0.134	0.082	-0.035	0.085	-0.028
	0.992	0.797	< 0.001	0.233	0.008	0.106	0.483	0.094	0.586

**Table 5:** Logistic regression analysis of expression levels of nine NER proteins in cases and controls.

NER proteins	Protein levels^∗∗∗^	Case *n* (%)	Control *n* (%)	Crude OR (95% CI)	Adjusted OR^∗^ (95% CI)
XPA	≥0.215	139 (41.3)	143 (50.2)	1.00 (ref)	1.00 (ref)
	<0.215	198 (58.7)	142 (49.8)	1.43 (1.04-1.97)	1.42 (1.03-1.96)
*P* ^∗∗^				0.026	0.031

XPB	≥0.576	145 (37.1)	143 (50.2)	1.00 (ref)	1.00 (ref)
	<0.576	192 (62.9)	142 (49.8)	1.33 (0.97-1.83)	1.33 (0.97-1.83)
*P* ^∗∗^				0.075	0.075

XPC	≥0.220	173 (51.3)	142 (49.8)	1.00 (ref)	1.00 (ref)
	<0.220	164 (48.7)	143 (50.2)	0.94 (0.69-1.29)	0.94 (0.69-1.29)
*P* ^∗∗^				0.707	0.710

XPD	≥0.363	152 (45.1)	143 (50.2)	1.00 (ref)	1.00 (ref)
	<0.363	185 (54.9)	142 (49.8)	1.23 (0.89-1.68)	1.23 (0.90-1.69)
*P* ^∗∗^				0.207	0.199

XPF	≥0.216	168 (49.8)	143 (50.2)	1.00 (ref)	1.00 (ref)
	<0.216	169 (50.2)	142 (49.8)	1.01 (0.74-1.39)	1.01 (0.73-1.38)
*P* ^∗∗^				0.936	0.971

XPG	≥0.381	193 (57.3)	143 (50.2)	1.00 (ref)	1.00 (ref)
	<0.381	144 (42.7)	142 (49.8)	0.75 (0.55-1.03)	0.75 (0.54-1.03)
*P* ^∗∗^				0.077	0.074

ERCC1	≥0.231	195 (57.9)	142 (49.8)	1.00 (ref)	1.00 (ref)
	<0.231	142 (42.1)	143 (50.2)	0.72 (0.53-1.06)	0.75 (0.52-1.06)
*P* ^∗∗^				0.068	0.059

DDB1	≥0.143	173 (51.3)	144 (50.5)	1.00 (ref)	1.00 (ref)
	<0.143	164 (48.7)	141 (49.5)	0.97 (0.71-1.33)	0.97 (0.71-1.33)
*P* ^∗∗^				0.841	0.853

DDB2	≥0.522	167 (49.5)	142 (49.8)	1.00 (ref)	1.00 (ref)
	<0.522	170 (50.5)	143 (50.2)	1.01 (0.74-1.39)	1.01 (0.73-1.38)
*P* ^∗∗^				0.947	0.959

NER = nucleotide excision repair; OR = odds ratio; CI = confidence interval. ^∗^Adjusted for age, sex, smoking, and alcohol status. ^∗∗^*P* value in trend test by continuous protein expression levels. ^∗∗∗^Expression levels by medians based on the median values of control subjects.

**Table 6:** Logistic regression analysis of mRNA expression levels of nine NER genes in cases and controls.

NER genes	Median levels^∗∗∗^	Case *n* (%)	Control *n* (%)	Crude OR (95% CI)	Adjusted OR^∗^ (95% CI)
*XPA*	≤19.93	91 (41.2)	86 (50.0)	1.00 (ref)	1.00 (ref)
	>19.93	130 (58.8)	86 (50.0)	0.86 (0.58-1.29)	1.37 (0.91-2.05)
*P* ^∗∗^				0.011	0.024

*XPB*	≤19.45	80 (36.2)	86 (50.0)	1.00 (ref)	1.00 (ref)
	>19.45	141 (63.8)	86 (50.0)	1.76 (1.17-2.64)	1.71 (1.13-2.59)
*P* ^∗∗^				< 0.001	< 0.001

*XPC*	≤21.29	118 (53.4)	86 (50.0)	1.00 (ref)	1.00 (ref)
	>21.29	103 (46.6)	86 (50.0)	0.86 (0.58-1.29)	0.86 (0.58-1.29)
*P* ^∗∗^				0.583	0.563

*XPD*	≤21.60	98 (44.6)	86 (50.0)	1.00 (ref)	1.00 (ref)
	>21.60	122 (55.4)	86 (50.0)	1.26 (0.84-1.88)	1.23 (0.82-1.84)
*P* ^∗∗^				0.063	0.088

*XPF*	≤21.88	92 (42.0)	86 (50.0)	1.00 (ref)	1.00 (ref)
	>21.88	127 (58.0)	86 (50.0)	1.38 (0.92-2.06)	1.37 (0.91-2.05)
*P* ^∗∗^				0.033	0.038

*XPG*	≤20.67	105 (47.5)	86 (50.0)	1.00 (ref)	1.00 (ref)
	>20.67	116 (52.5)	86 (50.0)	1.12 (0.75-1.67)	1.11 (0.74-1.67)
*P* ^∗∗^				0.518	0.489

*ERCC1*	≤20.24	107 (48.4)	86 (50.0)	1.00 (ref)	1.00 (ref)
	>20.24	114 (51.6)	86 (50.0)	1.07 (0.72-1.59)	1.05 (0.70-1.56)
*P* ^∗∗^				0.985	0.995

*DDB1*	≤18.24	112 (50.7)	86 (50.0)	1.00 (ref)	1.00 (ref)
	>18.24	109 (49.3)	86 (50.0)	0.97 (0.65-1.45)	0.96 (0.64-1.44)
*P* ^∗∗^				0.254	0.271

*DDB2*	≤19.83	125 (56.6)	86 (50.0)	1.00 (ref)	1.00 (ref)
	>19.83	96 (43.4)	86 (50.0)	0.76 (0.51-1.13)	0.74 (0.49-1.11)
*P* ^∗∗^				0.879	0.833

NER = nucleotide excision repair; HNSCCs = head and neck squamous cell carcinomas; OR = odds ratio; CI = confidence interval. ^∗^Adjusted for age, sex, smoking, and alcohol status. ^∗∗^*P* value in trend test by continuous mRNA expression levels. ^∗∗∗^Expression levels by medians based on the median values of control subjects. The expression levels of eight NER genes were calculated by ΔCt; the higher the ΔCt values represent the lower expression levels of the target mRNA.

## Data Availability

The NER proteins and mRNA data used to support the findings of this study are included within the article.

## Abbreviations

HNSCCs: Head and neck squamous cell carcinomas
NER: Nucleotide excision repair
HPVs: Human papillomaviruses
OR: Odds ratio
CI: Confidence interval
ROC: Receiver operating characteristic curve.

## Supplementary Materials

Supplementary 1 Supplementary Figure 1: stratified ROC curves by drinking status calculated in multivariate logistic models. (A) The AUC was insignificantly improved in never drinkers that included the effect of XPA expression levels (*P* = 0.404). (B) The AUC was significantly improved in former drinkers (*P* = 0.001). (C) The AUC was significantly improved in current drinkers (*P* = 0.001).

Supplementary 2 Supplementary Table 1: correlation between expression levels of nucleotide excision repair proteins.
