# Supplementary material for: Combining Phenotypes of Nucleotide Excision Repair Pathway to Predict the Risk of Head and Neck Squamous Cell Carcinomas in a Chinese Population
Source: Dis Markers. 2022 Sep 7;2022:4959737. doi: 10.1155/2022/4959737 (PMC9476247; doi:10.1155/2022/4959737)
Supplement: Supplementary 1 — Supplementary Figure 1: stratified ROC curves by drinking status calculated in multivariate logistic models. (A) The AUC was insignificantly improved in never drinkers that included the effect of XPA expression levels (P = 0.404). (B) The AUC was significantly improved in former drinkers (P = 0.001). (C) The AUC was significantly improved in current drinkers (P = 0.001). [file 4959737.f1.docx]

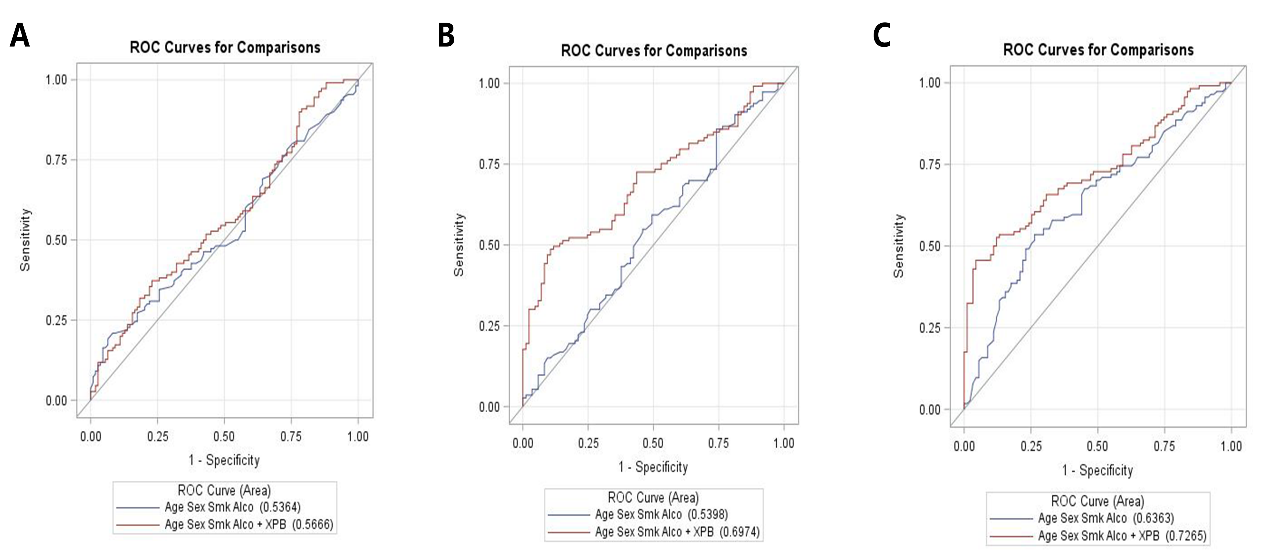


**Supplementary Fig. 1.** Stratified ROC curves by drinking status calculated in multivariate logistic models. **(A)** The AUC was insignificantly improved in never drinkers that included the effect of XPA expression levels (*P* = 0.404); **(B)** The AUC was significantly improved in former drinkers (*P* = 0.001); **(C)** The AUC was significantly improved in current drinkers (*P* = 0.001)
