# Supplementary material for: Combining Phenotypes of Nucleotide Excision Repair Pathway to Predict the Risk of Head and Neck Squamous Cell Carcinomas in a Chinese Population
Source: Dis Markers. 2022 Sep 7;2022:4959737. doi: 10.1155/2022/4959737 (PMC9476247; doi:10.1155/2022/4959737)
Supplement: Supplementary 2 — Supplementary Table 1: correlation between expression levels of nucleotide excision repair proteins. [file 4959737.f2.docx]

| **Table S1.** Correlation between expression levels of nucleotide excision repair proteins (n=622) | | | | | | | | | | | | |
| --- | --- | --- | --- | --- | --- | --- | --- | --- | --- | --- | --- | --- |
| Proteins | | Pearson correlation coefficients/*P* value | | | | | | | | | | |
|  |  | XPA | XPB | XPC | XPD | XPF | XPG | | ERCC1 | | DDB1 | |
| XPB | -0.094 | |  |  |  |  |  |  | |  | | |
|  | 0.019 | |  |  |  |  |  |  | |  | | |
| XPC | 0.079 | | 0.082 |  |  |  |  |  | |  | | |
|  | 0.050 | | 0.040 |  |  |  |  |  | |  | | |
| XPD | 0.207 | | -0.087 | -0.077 |  |  |  |  | |  | | |
|  | < 0.001 | | 0.031 | 0.054 |  |  |  |  | |  | | |
| XPF | 0.050 | | 0.046 | -0.050 | -0.032 |  |  |  | |  | | |
|  | 0.210 | | 0.247 | 0.217 | 0.428 |  |  |  | |  | | |
| XPG | 0.020 | | 0.035 | 0.079 | -0.021 | 0.214 |  |  | |  | | |
|  | 0.615 | | 0.378 | 0.050 | 0.603 | < 0.001 |  |  | |  | | |
| ERCC1 | -0.100 | | 0.119 | 0.063 | -0.115 | -0.086 | 0.021 |  | |  | | |
|  | 0.012 | | 0.003 | 0.117 | 0.004 | 0.032 | 0.606 |  | |  | | |
| DDB1 | 0.040 | | -0.040 | -0.056 | -0.021 | -0.021 | 0.072 | 0.077 | |  | | |
|  | 0.317 | | 0.319 | 0.161 | 0.597 | 0.600 | 0.072 | 0.056 | |  | | |
| DDB2 | -0.031 | | 0.027 | -0.024 | -0.007 | -0.115 | 0.016 | -0.007 | | -0.007 | | |
|  | 0.439 | | 0.495 | 0.552 | 0.864 | 0.004 | 0.694 | 0.859 | | 0.853 | |  |

| **Table S2.** Stratification analysis of expression levels of XPA of HNSCC cases by tumor sites | | |  |
| --- | --- | --- | --- |
| Variable | XPA (Mean ± SD, n = 337) |  |  |
| Tumor site |  |  | |
| Oral cavity | 0.194 ± 0.029 |  |  |
| Oropharynx | 0.201 ± 0.035 |  |  |
| Larynx/Hypopharynx | 0.199 ± 0.032 |  |  |
| *P** | 0.516 |  |  |
| HNSCCs = head and neck squamous cell carcinomas  ^*^*P* value in Wilcoxon rank-sum tests | | |  |
